# Trapping of the malaria vector *Anopheles gambiae *with odour-baited MM-X traps in semi-field conditions in western Kenya

**DOI:** 10.1186/1475-2875-5-39

**Published:** 2006-05-15

**Authors:** Basilio N Njiru, Wolfgang R Mukabana, Willem Takken, Bart GJ Knols

**Affiliations:** 1 International Centre of Insect Physiology and Ecology (ICIPE), Thomas Odhiambo Campus at Mbita Point, P.O. Box 30, Mbita, Kenya; 2 Department of Zoology, University of Nairobi, P.O. Box 30197 – 00100 GPO, Nairobi, Kenya; 3 International Atomic Energy Agency (IAEA), Agency's Laboratories Seibersdorf, A2444 Seibersdorf, Austria; 4 Laboratory of Entomology, Wageningen University and Research Center, P.O. Box 8031, 6700 EH Wageningen, The Netherlands

## Abstract

**Background:**

The successful development of odour-baited trapping systems for mosquitoes depends on the identification of behaviourally active semiochemicals, besides the design and operating principles of such devices. A large variety of 'attractants' has been identified in laboratory investigations, yet few of these increase trap catches in the field. A contained system, intermediate between the laboratory and open field, is presented and previous reports that human foot odour induces behavioural responses of *Anopheles gambiae *confirmed.

**Methods:**

The response of 3–5 day old female *An. gambiae *towards odour-baited counterflow geometry traps (MM-X model; American Biophysics Corp., RI) was studied in semi-field (screen house) conditions in western Kenya. Traps were baited with human foot odour (collected on socks), carbon dioxide (CO_2_, 500 ml min^-1^), ammonia (NH_3_), 1-octen-3-ol, or various combinations thereof. Trap catches were log (x+1) transformed and subjected to Latin square analysis of variance procedures.

**Results:**

Apart from 1-octen-3-ol, all odour baits caused significant (P < 0.05) increases in trap catches over non-baited traps. Foot odour remained behaviourally active for at least 8 days after collection on nylon or cotton sock fabric. A synergistic response (P < 0.001) was observed towards the combination of foot odour and CO_2_, which increased catches of these odours alone by 3.8 and 2.7 times, respectively.

**Conclusion:**

These results are the first to report behavioural responses of an African malaria vector to human foot odour outside the laboratory, and further investigation of fractions and/or individual chemical components of this odour complex are called for. Semi-field systems offer the prospect of high-throughput screening of candidate kairomones, which may expedite the development of efficient trap-bait systems for this and other African mosquito species.

## Background

Development of odour-baited trapping devices for biting insects remains a challenge for many important species, including African malaria vectors [1,2]. Such traps may find application in mosquito surveillance [3], risk assessment and forecasting [4], and/or be used *en masse *for population suppression and disease transmission reduction similar to trap-bait systems developed for tsetse flies [5-7]. There are three important components of trap development, namely the 'attractant', the physical trap design, and trapping mechanism used. A fourth set of essentials follows, namely the cost, applicability and acceptance of such devices by end-users in anticipated market sectors.

*Anopheles gambiae s.s*. is a highly anthropophilic mosquito, with a tendency to blood feed and rest inside houses [8]. Studies on semiochemicals affecting its host-seeking behaviour have intensified since the late 1980s [1], with the main aim to replace the Human Biting Catch (HBC). Some traps use whole human odour, like the CDC light trap placed next to an occupied bednet [9], the OBET [10,11] and Mbita traps [12-14]. However, humans vary substantially in their innate attractiveness towards *An. gambiae s.s*. [15,16], which has been attributed to the presence of allomonal effects of breath [17], variability in skin microfloral composition [18,19], or both. Identification of specific kairomones with key involvement in governing the host-seeking process has therefore been advocated [20].

Of the several hundreds of volatiles produced by humans [21,22], a fair number have been reported to elicit behavioural responses by *An. gambiae s.s*. These comprise commonly known kairomones like carbon dioxide [23-25], but also carboxylic fatty acids [26,27], oxo-carboxylic acids [28], ketones [29], phenols [29,30], L-lactic acid [31], and ammonia [32].

It remains speculative why attempts to reproduce laboratory studies under field conditions have been unsuccessful to date, although the substantial differences between olfactometer-based studies [26-31] and field-based trapping methods may be a prime cause for this. Alternatively, prolonged maintenance of mosquito strains under artificial laboratory conditions may result in distorted behaviour and responses to 'attractants' that would not be similar in nature. A third reason relates to the highly endophagic behaviour of *An. gambiae s.s*. that results in strong 'competition' between odour-baited traps and human hosts present in the indoor environment, arguably always in favour of humans expressing the full range of physical and chemical cues. Finally, and in contrast with laboratory studies, ambient climatic conditions may vary over space and time, and cause highly variable trap catches in the field.

The development and commercialization of traps based on counterflow geometry technology in the late 1990s by the American Biophysics Corporation has resulted in efficacy evaluation studies for a range of mosquito species in various geographical settings, including Africa [33-35]. Many of these evaluations inferred superiority of this technology over conventional traps such as the CDC light trap [36], which supports the view that trap design rather than the stimuli used affect the trapping efficiency.

In the present study, it was aimed to reproduce some previous laboratory findings under semi-natural conditions in western Kenya. In large outdoor cages (for a description see [37]) experimental counterflow geometry traps were deployed (see below), baited with human foot odour, CO_2_, NH_3_, 1-octen-3-ol, or various combinations thereof, as a first step to develop more appropriate research tools for anticipated open field studies.

## Materials and methods

### Mosquitoes

Two strains of *An. gambiae s.s.* mosquitoes were used. These originated either from Njage village, 70 km from Ifakara, South-East Tanzania, (maintained under laboratory conditions since April 1996) or from Mbita Point, Western Kenya (maintained since January 2001). Adult mosquitoes belonging to these strains were kept in 30× 30× 30 cm gauze-covered cages under ambient conditions at the Thomas Odhiambo Campus (00°25'S, 34°13'E), Nyanza Province, western Kenya, a field station of the International Centre for Insect Physiology and Ecology (ICIPE). They were maintained on a 6% glucose solution and provided with water on cotton wicks to increase relative humidity in cages. Mosquitoes were fed on a human arm for 10 min every three days. Eggs, laid on moist filter paper, were transferred into breeding trays and larvae developed up to pupal stage in water originating from Lake Victoria. Water was replaced at 1–2 day intervals. Larvae were fed 2–3 times daily on Tetramin^® ^fish food. Pupae were collected daily and transferred to adult holding cages containing sugar water and water-moistened cotton wicks. Further details on mosquito colony maintenance are reported elsewhere [37].

The age of females used in the experiments was 3–5 days and these were starved for 6 hrs before the experiments in a 1 L cup, covered by mosquito netting, and were offered water-moistened wicks only.

### Experimental procedures

**Figure 1:**
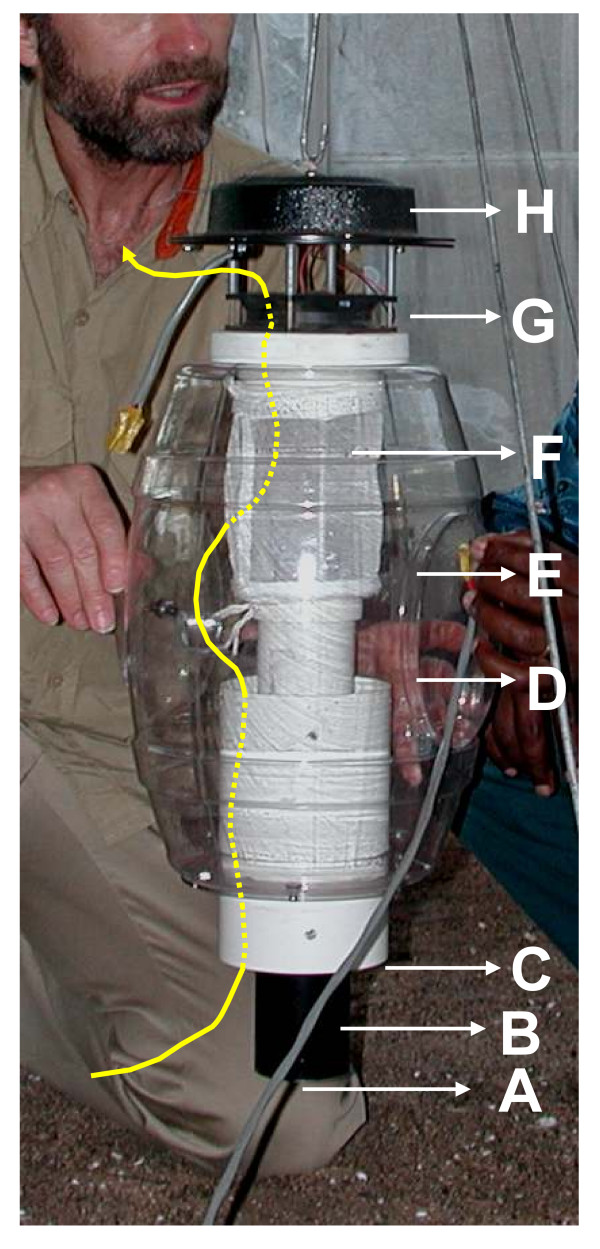
The MMX counterflow geometry trap (American Biophysics, RI, USA). A: Experimental odour outlet; B: Central tube; C: Mosquito entry point; D: Plastic transparent mosquito holding container; F: Computer fan pumping odour downward; G: Computer fan sucking air upward; H: Rain shield. The yellow arrow depicts the flow of air through the trap.

All experiments were conducted in a greenhouse (Cambridge Glass House Co. Ltd., UK) with a glass-panelled roof and gauze covered side walls. Inside, a layer of sand was put on the floor and a large mosquito-netting cage (10× 6× 2.5 m; mesh width 3 mm) was mounted. Two counter flow geometry traps (MM-X model; American Biophysics Corp., USA; Fig 1.)), were suspended 8 m from each other, with the odour outlet 15 cm above ground level. For a detailed description of the trap see [38]. Every test night, 200 experimental mosquitoes were released from the centre of the greenhouse at 19.00 hrs local time and trap catches collected at 07.00 hrs the following morning. Trap positions and treatments were randomised over experimental nights to avoid possible side effects.

### Experiments with foot odour

Foot odours were either collected from BNN (male African, age 34 yrs) or BGJK (male Caucasian, 35 yrs) on sock material by wearing them for 12 hrs (7 am – 7 pm) prior to the start of the experiments. Two types of fabric were used, being either nylon or cotton. Worn socks were inserted in the central black cylinder of the trap (see Fig. 1) and fitted in such way that the airflow was not obstructed. Dry, clean socks were used in the control traps. The weight increase (water/odours) between clean and worn nylon and cotton socks was (average ± SD of 4 days) 180 ± 29 mg and 670 ± 186 mg, respectively. Every day a new clean set of socks was used for the experiments (16 nights).

In a second series of experiments nylon socks were worn by both volunteers for a period of 12 hours only. The socks were subsequently used at 24 h intervals for trapping (7 pm – 7 am) until catch levels dropped to <5 mosquitoes per day for two consecutive days. Socks were not removed from the traps during the day (7 am -7 pm) and the outlet of the black pipe (Fig. 1) was covered with aluminium foil. Traps (including the socks) were put in a freezer (-5°C) for 20–30 min every morning to kill the mosquitoes caught the previous night.

### Other experimental odours

In a series of five further experiments (replicated over four nights each), traps baited with NH_3_, CO_2_, 1-octen-3-ol, foot odour (collected as described above) were compared against unbaited traps. Ammonia was diluted in distilled water to obtain various concentrations (0.1, 1, 10% (v/v) or undiluted, offered as 10 ml aliquots) offered from an open glass vial (aperture 1 cm) placed near the odour outlet in the central tube of the trap. CO_2 _was released in the central tube (at 500 ml/min) from a pressurised gas cylinder through 5 mm silicon tubing and controlled using a flow-meter. 1-Octen-3-ol was dispensed (~0.5 mg/h) from commercially available cartridges (American Biophysics, RI, USA) and placed in the central tube of the trap. A new cartridge was used on each test night.

In order to study the influence of various odours on trap catches, either alone or in combination, four traps were run simultaneously in the four corners of the greenhouse. Treatments and traps were randomised over positions to complete one block of a 4× 4 Latin square (4 test days). In three test series, foot odour and/or ammonia, carbon dioxide and/or ammonia, and foot odour and/or carbon dioxide were tested. Concentrations of volatiles were similar to those described above.

### Statistical analysis

Trap catches were log (n+1) transformed and subjected to Latin square analysis of variance [39]. A F-test significant at P < 0.05 was followed by a Least Significant Difference (LSD) *post hoc *test to sort out differences between treatment means.

## Results

When traps were baited with worn socks only, average (± SD) catches over eight test nights were 47 ± 21 for BNN and 73 ± 20 for BGJK (Table 1). Three out of four completed Latin square blocks yielded highly significant attraction to foot odour and in spite of high catches in the first block of BGJK, the conservative discriminatory levels needed when applying a double replicate of 2× 2 Latin square cross-over designs caused insignificance because of the control trap yielding 21 specimens in the first night. The overall effect of fabric (nylon or cotton) was not significant.

**Table 1:** MM-X trap catches (n = 200 released per night) of *An. gambiae s.s*. (Njage strain) for 4 trap nights when baited with worn socks of either nylon or cotton fabric worn by BNN or BGJK. Clean socks of the same fabric served as controls.

Person Day		Nylon	Control	Cotton	Control
BNN	1	53	2	93	2
	2	37	4	38	9
	3	34	1	41	4
	4	55	2	25	2
	F = 141.8; P < 0.001			F= 99.9; P < 0.001	
					
BGJK	1	75	21	74	1*
	2	98	5	58	1
	3	95	0	38	2
	4	59	2	84	6
	F = 12.1; ns			F = 76.3; P < 0.001	

* Catch for the first night excluded (9-0), with cold weather, strong winds and rain.

When evaluating the attractiveness of foot odours collected on socks worn for 12 hours only, unexpectedly high levels of residual activity were observed (Table 2). This effect was stronger for BGJK than for BNN. Nevertheless, two days after collection of foot odour of BNN, 39% (78/200) of released mosquitoes were caught, whereas BGJK's residual odours still trapped nearly 27.5% (55/200) of the females released as long as seven days after initial odour collection.

**Table 2:** MM-X trap catches (n= 200 released per night) of *An. gambiae s.s*. (Njage strain) when baited with a nylon (BGJK) or cotton sock (BNN) worn for 12 hr only and then used at 24 hr intervals afterwards. Clean socks of the same fabric served as controls.

Hour*	Nylon	Control	Cotton	Control
0–12	59^b^	2	73	2
24–36	97	0	31	0
48–60	40	2	78	0
72–84	59	1	48	0
96–108	33	0	5	0
120–132	43	0	13	0
144–156	55	2	6	1
168–180	55	1	7	0
192–204	28	1	7	1

* Starting from the time the socks were removed; **Socks from last day Table 1.

Albeit at lower levels, NH_3 _when offered in various concentrations, raised trap catches significantly (at 0.1, 1 and 10%; Table 3A) except when offered undiluted. No clear dose-response effect was observed. As expected, CO_2_, a well-known kairomone, elicited strong behavioural responses, with an average (± SD) of 110 ± 26 females collected (~55% of the number released) per night. In contrast, 1-octen-3-ol at 0.5 mg/hr did not raise catches significantly over those collected by the control trap (Table 3C). When foot odour was combined with NH_3 _(10% v/v) catches remained significantly different from the control trap, though on average similar to catches with foot odour only (Table 3D). However, when CO_2 _was added to this blend, a sharp and significant increase in catches was observed, with an average (± SD) catch of 181 ± 43 females (~91% of the number released; Table 3E). In fact, it was noticed that, even in the absence of sugar sources in the greenhouse, mosquitoes managed to survive between test periods (12 hrs), as catches in this series twice exceeded the 200 insects released during the experimental night.

**Table 3:** MM-X trap catches (n = 200 released per night) of *An. gambiae s.s*. (Mbita strain) for 4 trap nights when baited with (A) NH_3_(0.1, 1, 10 or 100%), (B) CO_2_(500 ml/min), (C) 1-octen-3-ol, (D) foot odour (FO; from BNN) + NH_3_(10%), (E) foot odour (FO; from BNN) + CO_2_(500 ml/min) + NH_3_. Controls were unbaited traps.

Expt.	Day	NH_3 _(0.1%)	Control	NH_3 _(1.0%)	Control
A	1	18	1	29	3
	2	11	1	15	3
	3	7	1	17	2
	4	5	2	18	1
	F = 19.2; P < 0.05			F= 69.4; P < 0.001	
		NH_3 _(10%)	Control	NH_3 _(100%)	Control
	
	1	31	2	22	3
	2	11	1	14	2
	3	25	2	11	5
	4	16	2	3	2
	F = 1396.8; P < 0.001			F= 6.58; ns	
B, C		CO_2_	Control	1-octen-3-ol	Control
	
	1	122	1	1	1
	2	119	1	5	1
	3	127	1	2	4
	4	71	0	20	5
	F = 7977.3; P < 0.001			F = 11.9; ns	
D, E		FO+ NH_3_	Control	FO+NH_3_+CO_2_	Control
	
	1	120	1	190	5
	2	73	1	205	2
	3	82	0	210	0
	4	65	3	118	0
	F = 296.6; P < 0.001			F= 81.9; P < 0.001	

When four traps were tested simultaneously, and three of these contained odour baits, trap catches with NH_3 _were similar to those of the control trap and significantly reduced catches when combined with foot odour over foot odour alone (Table 4A). In a further series (Table 4B), NH_3 _again did not increase trap catches significantly, nor did it affect catches when combined with carbon dioxide. As observed in previous experiments (Table 3E), a strong increase in catches when foot odours were combined with CO_2 _was expected. Table 4C shows not only that foot odour and CO_2 _alone caused significant increases in catches over control traps, but also that the combination of the two led to a significant and synergistic increase in catch levels (i.e. the combination of the two yielded significantly higher catches than either or the sum of the two baits alone).

**Table 4:** 4 × 4 Latin square experiments using various combinations of odours in combination with MM-X traps. Catches of *An. gambiae s.s*. (Mbita strain) for 4 trap nights (200 released per night), including totals caught are shown. FO = Foot odour (from BNN). Totals in the same column not followed by the same letter are significantly different at P < 0.05.

Treatment	Day	1	2	3	4	Total	F	P
(A)								
Control		4	2	4	3	13 a	18.7	<0.001
FO		16	69	39	28	152 b		
NH_3_*		6	8	5	8	27 a		
FO+NH_3_		9	29	7	15	60 c		
								
(B)								
Control		9	11	11	8	39 a	50.1	<0.001
CO_2_**		35	78	51	56	220 b		
NH_3_*		12	14	6	7	39 a		
CO_2_+NH_3_		62	62	34	53	211 b		
								
(C)								
Control		11	6	8	5	30 a	34.9	<0.001
FO		14	21	31	27	93 b		
CO_2_**		35	14	31	49	129 b		
FO+CO_2_**		77	98	105	71	351 c		

*10% (v/v); ** 500 ml/min

**Figure 2:**
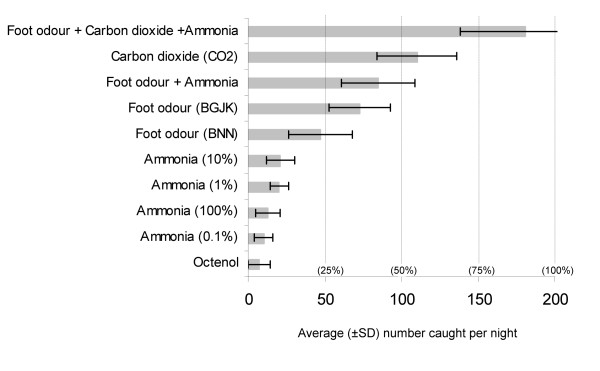
Average catch levels (± SD) for the different odours and combinations tested. Percentages indicate the proportion of overall number of mosquitoes released.

An overview of the experimental baits and catch levels is shown in Figure 2. Overall, catch levels of both strains of *An. gambiae s.s*. when responding to foot odour, appeared similar.

## Discussion

In the early 1990s, foot odour was first incriminated as influencing the selection of biting sites by *An. gambiae s.s*. on humans [40]. Subsequently it was found that this mosquito also responds strongly to Limburger cheese volatiles, reminiscent of human foot odour, in laboratory bioassays [26,41,42]. Repeated efforts to show similar effects in the field, when baiting various trap models and electric nets [43] with these complex odours were disappointing [44] and not reported (Knols and Mboera, unpublished data). However, in the present study it was clearly demonstrated that foot odours, when collected on either cotton or nylon sock fabric, can significantly increase trap catches and thus confirm earlier laboratory findings [40]. It is noteworthy that the residual activity of these emanations spanned several days, corroborating earlier studies claiming that previously occupied houses or worn clothing attracted mosquitoes several days after having been vacated or worn, respectively [45]. These findings clearly indicate that as yet unknown kairomones with low volatility are present in foot odour. Progression in the identification of active fractions of foot odour is currently underway (A. Hassanali, *personal communication*).

Ammonia has been demonstrated to elicit behavioural and sensory physiological responses of many haematophagous arthropods including Afrotropical Anopheles [46-48]. Either on its own, but particularly when combined with lactic acid and a blend of carboxylic fatty acids, laboratory assays confirmed the kairomonal effect of this chemical [48]. The current findings show a mildly attractive effect of ammonia alone, but did not indicate an additive or synergistic effect when combined with either foot odour or CO_2_. It should be noted though that the high volatility of this compound in an aqueous solution could have caused only short-term effects. Alternatively, with ammonia already naturally present in foot odour additional ammonia did not cause any measurable behavioural effect. A slow-release system, analogous to the continuous production of ammonia on the human skin through microbial breakdown of urea and amino acids [46], might improve trap catches.

Carbon dioxide, in all experiments, dramatically increased trap catches. Regretfully, the use of this kairomone either in gaseous form or as dry ice remains a major hurdle for its inclusion in trapping devices in the tropics. Although alternative delivery systems have been developed (such as the catalytic combustion of propane), these still depend on costly gas tanks not widely available. Although it has been argued that anthropophilic vectors cannot depend solely on this chemical to locate humans [1], CO_2 _clearly synergises responses to other human-specific substances. Although mosquito surveillance programmes may use CO_2_-baited devices, widespread use of traps at household level will ultimately require identification of potent bait without the need for CO_2_. At present, kairomones present in foot odour seem to offer the best promise for this. It should be noted though, that semi-field systems cannot replace field studies and that verification of findings should always take place. Similarly, although this trapping system has proven useful for field sampling of mosquitoes (Yu Tong Qiu et al., unpublished data from field studies in the Gambia), it should be considered primarily as an experimental tool to evaluate candidate kairomones and not directly as a replacement for existing sampling tools for adult anophelines.

## Conclusion

In spite of decades of research, no effective trapping system for *An. gambiae *is currently available. Lack of appropriate trapping mechanisms and systems that enable rigorous evaluation have seriously hindered progress. The current work has demonstrated the usefulness of contained semi-field environments to rapidly evaluate the potency of bait systems and confirm laboratory findings. Foot odour was shown for the first time to elicit behavioural responses in this system, and acted synergistically with carbon dioxide. The role of ammonia remains less clear, and merits further evaluation.

## Authors' contributions

BNN and WR conducted the experimental work and drafted the manuscript. BGJK conceived of the studies and wrote the final version of the manuscript in collaboration with WT.
